# Emotional control and factors differentiating it in the adult population of Poland during the COVID-19 pandemic

**DOI:** 10.3389/fpsyg.2023.1225698

**Published:** 2023-06-22

**Authors:** Anna Głogowska-Gruszka, Agata Wypych-Ślusarska

**Affiliations:** ^1^Department of Chronic Diseases and Civilization-related Hazards, Faculty of Public Health in Bytom, Medical University of Silesia, Bytom, Poland; ^2^Department of Epidemiology, Faculty of Public Health in Bytom, Medical University of Silesia, Bytom, Poland

**Keywords:** emotional control, anger, anxiety, depression, stress, COVID-19

## Abstract

**Introduction:**

The public health crisis related to the COVID-19 pandemic has had a negative impact on the mental health of both individuals and entire populations. The source of stress was not only the fear of getting sick, but also the restrictions introduced, such as: mass lockdown, the need to maintain social distance, quarantine or the mandatory use of personal protective equipment. Their introduction and maintenance caused various emotional reactions which often resulted in undesirable behavior leading to infections spreading.

**The aim of the study:**

The aim of the study was to analyze the level of emotional control depending on selected factors related to the pandemic and the introduced restrictions.

**Materials and methods:**

The study covered 594 adult Poles. To evaluate knowledge about COVID-19 and attitudes toward the implemented restrictions, the questionnaire prepared by the authors was used. To determine the level of control of anger, depression and anxiety the Courtauld Emotional Control Scale (CECS) was used, and to estimate the level of perceived stress the Perceived Stress Scale (PSS-10) was applied.

**Results:**

In the entire analyzed group, the general level of emotional control was 51.82 ± 12.26, with anxiety being the most suppressed emotion (17.95 ± 4.99), whereas the least suppressed emotion was anger (16.35 ± 5.15). The average stress level in the studied group was 20.5 ± 5.3. The level of perceived stress did not differentiate the level of emotional control. It was found that the higher level of the knowledge about the pandemic and methods of prevention, the higher emotional control, especially in the anxiety subscale (high level of knowledge – 18.26 ± 5.36 vs. low level of knowledge - 15.09 ± 3.6; *p* = 0.02). People reporting difficulties in reconciling remote work with home duties were less able to control anger (14.63 ± 4.98) than people without such problems (16.71 ± 4.12; *p* = 0.007).

**Conclusion:**

Proper education improving knowledge about COVID-19 and methods of prevention may enhance the control of emotions in the population. Possible future preventive measures aimed at limiting the spread of SARS-CoV-2 infections or other infectious diseases should also take into account possible excessive mental burden caused by private and professional duties.

## Introduction

The public health crisis related to the COVID-19 pandemic has had a negative impact on the mental health of both individuals and entire populations. Numerous studies conducted so far have indicated that the COVID-19 pandemic was a significant source of stress, both for the general population and various social or professional groups surveyed. It was also associated with a higher incidence of depression, anxiety, PTSD and sleep problems (post-traumatic stress disorder) (Kumar and Nayar, 2020; Dragan et al., 2021; Li et al., 2021; O’Connor et al., 2021; Fountoulakis et al., 2022; Luo et al., 2022). Initially, the main source of stress for the general public was the fear of being infected and fear for the health and life of loved ones. To protect people from the new disease, the governments of almost all countries have introduced various restrictions, such as closing state borders, mass lockdown, the need to maintain social distance, prohibition of group gatherings, quarantine or the mandatory use of personal protective equipment. The main purpose of these measures was to limit the spread of infections. The introduced restrictions resulted in large, unprecedented changes in everyday life, the way of working, teaching, learning and social functioning. Often, like sadly in the case of many people, they contributed to the loss of jobs and the deterioration of their financial situation (Vinkers et al., 2020; Fountoulakis et al., 2022).

Initially, due to the common fear of falling ill with an unknown, new disease, the introduction of restrictions met with great approval and understanding which, as they were maintained for longer, decreased and caused various negative emotional reactions (powerlessness, discouragement, sadness, anger, fear, anxiety, etc.), which often translated into denying the danger of the pandemic, undermining the sense of the introduced restrictions, rebellion in the form of deliberate non-compliance with them or spreading conspiracy theories about the pandemic or coronavirus vaccines (Czeisler et al., 2020; Hagen et al., 2022; Kim et al., 2022; Szuster et al., 2022; Turska-Kawa and Pilch, 2022; Zhang et al., 2022).

In situations where negative emotions can come to the fore, especially if they can translate into non-compliance with anti-epidemic recommendations, it seems necessary to identify factors that may affect and modify their control. On one hand, expressing negative emotions is a beneficial phenomenon which is recommended in many forms of psychotherapy, as their long-term suppression may become the basis of many psychosomatic disorders (Doliński, 2006; Juczyński, 2012; Kim et al., 2022). On the other hand, uncontrolled expression of negative emotions, especially anger and fear, may turn into aggression directed not only toward loved ones, but also other people, e.g., non-compliance with the anti-epidemic restrictions (Abadi et al., 2021). This, in turn, can make it harder to fight the spread of infection.

The aim of the study was to analyse the level of emotional control depending on selected factors related to the COVID-19 pandemic and the introduced restrictions, such as personal experience related to the pandemic, the use of preventive measures, the subjective feeling of stress during the pandemic, and knowledge about the pandemic.

## Materials and methods

### Study design

In the period from March to June 2021 (during the third wave of the pandemic in Poland), a cross-sectional epidemiological study on a group of adult Poles was conducted using the questionnaire designed by authors, Courtauld Emotional Control Scale (CECS) and the Perceived Stress Scale (PSS-10). These questionnaires were distributed using the Internet surveying technique CAWI (Computer Assisted Web Interview). This study was a part of another larger survey on experienced emotions related to the COVID-19 pandemic, from which it was spun off in the end of April 2021 and from that time on conducted independently.

The survey was fully anonymous and voluntary, and respondents were informed about it in the initial instructions for the survey. Respondents could opt out of the survey at any time. Inclusion criterion constituted a consent of respondent for filling out the questionnaire and age above 18. The upper age limit was not defined. Non-probability sampling technique, where subjects of the study recruit future subjects from among their acquaintances (snowball sampling), was applied. During the study 629 filled out questionnaires were collected. Twenty nine questionnaires were excluded from the analysis due to lack of completeness and failure of meeting the subjects’ age criterion. Only six questionnaires were filled out by people with vocational or elementary educational level. Due to the small number of these respondents, it was decided to exclude these questionnaires from the analysis as well. Finally, the group of respondents included 594 persons, including 468 women (78.8%).

### Research tools

The author’s questionnaire collected the basic demographic data (gender, age, educational level, marital status, place of residence, professional status, having minors under surveillance, financial status), information on being infected with the SARS-CoV-19 virus, possible hospitalization for this reason, being in quarantine or experiencing the death of a loved one due to COVID-19. The respondents were also asked whether they lost their jobs, their earnings or working hours were reduced, they had to work more than before the pandemic or they switched to remote work due to the introduced restrictions. The questions in the author’s questionnaire concerned also the issue of vaccination and the use of preventive measures by the respondents (wearing masks, keeping distance, hand disinfection, disinfection of purchased goods or leaving them in “quarantine”). With the questions: “Is COVID-19 contagious?,” “How is COVID-19 transmitted?,” “Can pets spread COVID-19 to human?,” “Can COVID-19 be spread by insects bites?,” “How can we protect ourselves from COVID-19?,” “Is there a treatment to remove the cause of COVID-19?” the level of knowledge about the COVID-19 pandemic and its prevention was verified. One point was awarded for each correct answer. Respondents could score a maximum of 9 points. It was assumed that people who scored 8–9 points had high knowledge about COVID-19, those with 5–7 points had average knowledge, and those who scored 4 or less had low knowledge about COVID-19.

The Courtauld Emotional Control Scale (CECS), in the version compatible with the Polish adaptation by Juczyński, was used. This tool contains 21 sentences that are divided into 3 subscales. Each of them contains seven statements that concern the manner of showing anger, depression, and fear. The scale is designed to test adults, both healthy ones and patients, and it serves to measure respondents’ control of anger, anxiety, and depression in difficult life situations. By marking the most suitable answer, respondents assess how often they express emotions in a way provided in the questionnaire on a 4-point scale from “almost never” - 1 point to “almost always” - 4 points. For each of the subscales, results are calculated separately. The sum of the results in each of the subscales ranges within 7–28 points. After summing the results of all three subscales, a general coefficient of emotional control is obtained, which determines the researched person’s conviction about their ability to control their reactions in a situation in which they experience the negative emotions. The total coefficient is in the range of 21–84 points. The higher the score, the more suppressed the emotions are. The reliability (Cronbach’s alpha) of the Polish version of the CECS is 0.80 for anger control; 0.77 for depression control; 0.78 anxiety control, and 0.87 for the general coefficient of emotional control (CECS) (Juczyński, 2012).

The 10-item Perceived Stress Scale (PSS-10), developed by S. Cohen for measurement of self-perceived stress related to someone’s own situation, was used. Each item is scored on a 5-point Likert scale (0-never; 4-very often). The total score ranges between 0 and 40. Higher scores reflect high stress levels. Cronbach’s alpha of the Polish version of the PSS-10 is 0.86 (Juczyński and Ogińska-Bulik, 2012).

### Statistical analysis

The statistical analyses were performed using the TIBCO Statistica 13.3 programme. Demographic characteristics of respondents and quality data were summarized in absolute numbers and percentage. Due to low number of respondents who declared divorced/widowed both these groups were merged. The following three categories of occupational activity were defined: employed (persons who at the time of the survey were occupationally active and had a job), students, and unemployed (respondents who at the time of the survey were not occupationally active and were not students).

The results of the Courtauld Emotion Control Scale (CECS), each subscale of the CECS and PSS-10 were demonstrated as mean values with standard deviation (SD). The Shapiro–Wilk test was applied to check normality. Distribution of all quantitative data appeared to diverge from the normal pattern, therefore methods of non-parametric statistics were used (Mann –Whitney U test and the Kruskal-Wallis test with post-hoc tests). The analysis of significance of the differences between the mean values in the compared groups was performed in observance with the rules of the chosen test. Correlation between qualitative variables were calculated using the rho-Spearman coefficient which measures the strength and direction of correlation between variables. In all of the analyses, the results were accepted as significant in cases when the probability value p was smaller than the accepted significance level 0.05 (*p* < 0.05).

## Results

In our study, respondents of the female gender constituted over three quarters of the surveyed group. Almost 40% of the respondents were at the age of 18–29. The average age of the respondents was 36.4 (SD = 13.3). The majority of respondents lived in cities. Three quarters of the respondents had higher education. Most of the respondents were employed. One out of five people reported having at least one chronic disease. More than half of the respondents assessed their financial situation as good. Detailed characteristics of the respondents is given in Table 1.

**Table 1:** Sociodemographic characteristics of the analyzed group.

Variables	*n* (%)
Gender	
Female	468 (78.8%)
Male	126 (21.2%)
Age (in years)	
18–29	236 (39.7%)
30–39	130 (21.9%)
40–49	150 (25.3%)
>50	78 (13.1%)
Place of residence	
City	520 (87.5%)
Village	74 (12.5%)
Educational level	
Middle	154 (25.9%)
Higher	440 (74.1%)
Marital status	
Married	274 (46.1%)
Never married	286 (48.2%)
Divorced/widowed	34 (5.7%)
Professional activity	
Employed	386 (65.0%)
Student	140 (23.6%)
Professionally inactive	68 (11.4%)
Chronic diseases	
Yes	122 (20.5%)
No	472 (79.5%)
Minors under surveillance	
Yes	243 (40,9%)
No	351 (59.1%)
Financial status	
Very good	102 (17.2%)
Good	302 (50.8%)
Mediocre	164 (27.6%)
Bad and very bad	26 (4.4%)

The overall result of the Courtauld Emotional Control Scale (CECS) in the entire analyzed group of adults was 51.82 ± 12.26, with fear being the most suppressed emotion (17.95 ± 4.99), whereas the least suppressed emotion was anger (16.35 ± 5.15). For suppression of depression the entire group scored 17.52 points (SD = 4.74). There was no difference in emotional control between women and men, both in general coefficient (CECS) and all three subscales. Similarly, place of residence, educational level, professional activity, and financial status did not differentiate the general level of emotional control in general coefficient (CECS) and all three subscales.

The general level of emotional control was not differentiated by age, although significant differences were found in the control of anger and depression, depending on the age of the respondents. *Post hoc* tests revealed that people aged 30–39 were significantly less able to control anger and depression compared to those aged 18–29 (respectively: *p* = 0.016, *p* = 0.05) and to control depression compared to respondents aged over 50 (*p* = 0.03). Married people inhibited anger (15.62 ± 5.13) and depression (16.88 ± 4.81) to a lesser extent, especially in comparison to divorced and widowed people (respectively: 18.36 ± 4.40; *p* = 0.04; 19.53 ± 5.06; *p* = 0.04). Statistical analysis also showed that people who had minors under surveillance suppressed anger in a significantly lower manner compared to those without children (respectively: 15.60 ± 5.11 vs. 16.87 ± 5.13; *p* = 0.03). Detailed data on the emotional control in the studied group, depending on the sociodemographic characteristics, are presented in Table 2.

**Table 2:** Emotional control of the studied group depending on sociodemographic data.

	General coefficient CECS		Anger suppression		Depression suppression		Anxiety suppression	
	Mean value ±SD	*p* value	Mean value ±SD	*p* value	Mean value ±SD	*p* value	Mean value ±SD	*p* value
**Gender**								
Women	51.34 ± 12.39	0.1	16.13 ± 5.15	0.1	17.41 ± 4.83	0.3	17.80 ± 5.09	0.3
Men	53.60 ± 11.70		17.19 ± 5.10		17.92 ± 4.36		18.49 ± 4.57	
**Age (in years)**								
18–29	53.14 ± 11.45	0.07	17.25 ± 5.09	**0.02**	18.04 ± 4.53	**0.03**	17.85 ± 4.93	0.5
30–39	48.85 ± 12.37		14.89 ± 5.27		16.35 ± 4.93		17.60 ± 4.70	
40–49	50.87 ± 11.66		15.92 ± 4.84		16.99 ± 4.41		17.96 ± 4.98	
>50	54.64 ± 14.63		16.92 ± 5.28		18.90 ± 5.21		18.82 ± 5.70	
**Place of residence**								
City	51.65 ± 12.40	0.5	16.32 ± 5.23	0.6	17.54 ± 4.79	0.9	17.80 ± 4.99	0.1
Village	53.00 ± 11.35		16.59 ± 4.63		17.38 ± 4.35		19.03 ± 4.95	
**Educational level**								
High	51.36 ± 12.26	0.3	16.07 ± 5.05	0.6	17.30 ± 4.66	0.9	17.99 ± 5.00	0.1
Middle	53.14 ± 12.25		17.17 ± 5.38		18.13 ± 4.92		17.84 ± 4.99	
**Marital status**								
Never married	52.63 ± 11.43	0.08	16.82 ± 5.17	**0.02**	17.89 ± 4.55	**0.03**	17.92 ± 4.85	0.9
Married	50.40 ± 12.89		15.62 ± 5.13		16.88 ± 4.81		17.91 ± 5.13	
Divorced/widowed	55.06 ± 11.76		18.36 ± 4.40		19.53 ± 5.06		18.59 ± 5.21	
**Professional activity**								
Student	53.37 ± 10.56	0.4	17.34 ± 4.80	0.1	18.06 ± 4.65	0.5	17.97 ± 5.02	0.9
Employed	51.43 ± 12.36		16.13 ± 5.12		17.41 ± 4.66		17.89 ± 4.97	
Professionally inactive	50.85 ± 14.79		15.59 ± 5.88		17.00 ± 5.36		18.26 ± 5.18	
**Chronic diseases**								
Yes	50.43 ± 13.48	0.4	15.62 ± 5.29	0.2	17.05 ± 5.15	0.6	17.75 ± 5.10	0.7
No	52.18 ± 11.93		16.54 ± 5.11		17.64 ± 4.63		18.00 ± 4.97	
**Minors under surveillance**								
Yes	50.78 ± 12.99	0.2	15.60 ± 5.11	**0.03**	17.10 ± 4.98	0.2	18.07 ± 5.23	0.5
No	52.54 ± 11.71		16.87 ± 5.13		17.81 ± 4.55		17.86 ± 4.83	
**Financial status**								
Very good	52.86 ± 12.50	0.2	16.71 ± 5.39	0.7	17.33 ± 4.94	0.09	18.82 ± 4.93	0.2
Good	50.42 ± 11.79		15.95 ± 4.85		16.97 ± 4.55		17.50 ± 5.03	
Mediocre	53.44 ± 12.95		16.79 ± 5.55		18.50 ± 4.76		18.15 ± 5.07	
Bad and very bad	53.77 ± 11.66		16.92 ± 5.27		18.38 ± 5.24		18.46 ± 4.03	

SD – standard deviation; Statistically significant differences have been marked in bold.

In our study, 12.8% of the respondents admitted that they had been infected with COVID-19. Out of this group, only 6 people were hospitalized. The conducted statistical analyses did not show any significant impact of the COVID-19 infection or COVID-19-related hospitalization on the level of emotional control of the respondents. It was also reported that 18.2% of respondents were in quarantine at least once. These people were characterized by lower suppression of anxiety than respondents who were not subject to such an obligation (16.93 ± 4.88 vs. 18.17 ± 4.98, *p* = 0.02). Nearly 42% of the survey participants admitted that they had personally known someone who died from COVID-19. These people obtained a significantly higher general coefficient CECS (53.41 ± 12.40), as well as higher rates of anger suppression (17.05 ± 5.14) and depression suppression (18.03 ± 4.83) compared to those who did not lose a loved one due to COVID-19 (*p* < 0.05).

Respondents were also asked about the impact of the pandemic on professional issues. It was reported that 6.0% of respondents lost their jobs due to the pandemic or were forced to close their businesses. It was also found that 10.4% of the survey participants were affected by limiting activity of company in which they were employed or working part-time. The same number of people were affected by the reduction in wages. In turn, 8.4% of respondents admitted that they work more than before the pandemic. Less than 20% of respondents admitted that they faced difficulties related to the need to reconcile work and/or remote learning with home duties, including childcare. From the group of factors related to professional issues, losing a job and working more than before the pandemic were associated with significantly lower suppression of anxiety, but did not modify the suppression of anger and depression or the general index of emotional control. In turn, people experiencing difficulties in reconciling remote work with home duties achieved a significantly lower value of the general coefficient of emotional control (CECS) compared to people who did not report such difficulties (49.14 ± 11.53 vs. 52.38 ± 12.36, *p* = 0.01). Particularly large differences were visible in the anger suppression subscale. Detailed data on emotional control, depending on the difficulties experienced during the pandemic are presented in Table 3.

**Table 3:** The level of emotional control in the studied group depending on the selected experiences related to the COVID-19 pandemic.

	General coefficient CECS		Anger suppression		Depression suppression		Anxiety suppression	
	Mean value ±SD	*p* value	Mean value ±SD	*p* value	Mean value ±SD	*p* value	Mean value ±SD	*p* value
COVID-19 infection								
Yes (*n* = 76)	51.82 ± 12.42	0.9	17.00 ± 5.30	0.2	17.39 ± 4.94	0.8	17.42 ± 4.82	0.3
No (*n* = 518)	51.83 ± 12.23		16.27 ± 5.12		17.55 ± 4.71		18.02 ± 5.01	
Hospitalization due to COVID-19								
Yes (*n* = 6)	47.67 ± 4.59	0.4	16.33 ± 1.37	0.9	15.33 ± 2.25	0.1	16.00 ± 2.68	0.2
No (*n* = 70)	52.52 ± 12.87		16.31 ± 5.40		18.13 ± 5.04		18.08 ± 5.38	
Being in quarantine								
Yes (*n* = 108)	50.44 ± 12.21	0.2	16.04 ± 4.97	0.5	17.48 ± 4.88	0.9	16.93 ± 4.88	**0.02**
No (*n* = 486)	52.14 ± 12.24		16.43 ± 5.19		17.54 ± 4.70		18.17 ± 4.98	
Death of a loved one due to COVID-19								
Yes (*n* = 249)	53.41 ± 12.40	**0.006**	17.05 ± 5.14	**0.005**	18.03 ± 4.83	**0.02**	18.33 ± 4.95	0.08
No (*n* = 345)	50.60 ± 11.99		15.85 ± 5.12		17.13 ± 4.63		17.62 ± 4.98	
Loss of job/closure of business								
Yes (36)	49.00 ± 11.32	0.1	16.44 ± 4.59	0.7	16.94 ± 4.96	0.6	15.61 ± 4.38	**0.03**
No (558)	52.00 ± 12.32		16.35 ± 5.19		17.56 ± 4.73		18.10 ± 4.99	
Limitation of job activities / reduction of work hours								
Yes (*n* = 62)	51.97 ± 10.14	0.9	16.97 ± 4.89	0.3	17.74 ± 4.83	0.7	17.26 ± 4.22	0.2
No (*n* = 532)	51.80 ± 12.50		16.28 ± 5.19		17.50 ± 3.82		18.02 ± 5.06	
Increased workload								
Yes (50)	48.60 ± 9.77	0.2	15.52 ± 4.27	0.4	16.64 ± 3.98	0.3	16.44 ± 3.82	**0.02**
No (544)	52.12 ± 12.44		16.43 ± 5.23		17.60 ± 4.80		18.09 ± 5.07	
Reduction of earnings								
Yes (62)	52.48 ± 14.37	0.7	17.19 ± 5.30	0.3	17.61 ± 5.50	0.9	17.68 ± 5.22	0.7
No (532)	51.74 ± 12.02		16.25 ± 5.14		17.51 ± 4.65		17.98 ± 4.97	
Remote work								
Yes (160)	51.27 ± 10.91	0.6	15.87 ± 4.79	0.3	17.22 ± 4.21	0.4	18.17 ± 4.47	0.7
No (434)	52.02 ± 12.74		16.53 ± 5.28		17.63 ± 4.92		17.87 ± 5.17	
Difficulties in reconciling remote work/learning with home duties								
Yes (102)	49.14 ± 11.53	**0.01**	14.63 ± 4.99	**0.007**	17.12 ± 4.38	0.4	17.39 ± 4.73	0.2
No (492)	52.38 ± 12.36		16.71 ± 5.12		17.60 ± 4.81		18.07 ± 5.04	

SD – standard deviation; Statistically significant differences have been marked in bold.

A quarter of the surveyed participants admitted that they had been vaccinated against the SARS-CoV-2 virus. These people were characterized by a significantly higher level of depression suppression (18.17 ± 4.11) compared to those who had not been vaccinated (17.32 ± 4.57; *p* = 0.04). Differences in overall CECS score and suppression of anger and anxiety between vaccinated and unvaccinated subjects were not statistically significant. Out of the group of people who were not vaccinated at the time of the study, almost half declared their willingness to be vaccinated as soon as possible. These people obtained higher values of both the general CECS emotion control coefficient and in all subscales, but the differences were not statistically significant. Over 93% of respondents admitted that they always wear masks in public places, none of the respondents declared that they never wear masks in such places. It was reported that 67.7% of participants declared wearing a mask at all times in public places, such as streets, parks, etc. The frequency of using masks in public outdoor and indoor places did not differentiate the level of emotional control in terms of any of the analyzed factors.

The obligation to maintain social distance in public spaces was always or almost always obeyed by 63.3% of respondents. In the case of outdoor public places, social distancing was always or almost always observed by slightly more than half of the respondents. People who never respected social distancing, both indoors and outdoors, had lower average values of all indicators of emotional control, but only in the case of anger suppression, the existing differences between those, who kept their distance in outdoor public places and those who never did so, were statistically significant (*p* = 0.03). Almost 60% of respondents declared that they always or almost always disinfect their hands before entering public spaces. The use of this prophylactic measure did not differentiate either the general coefficient of emotional control or anger suppression, depression suppression, and anxiety suppression. Similarly, the level of emotional control was not differentiated by the disinfection or leaving purchased goods in “quarantine,” which was always or almost always done by 18.2% of participants. Detailed data on the level of emotional control depending on the frequency of using various preventive measures limiting the spread of SARS-CoV-2 infections are presented in Table 4.

**Table 4:** Level of emotional control depending on application of prophylactic measures.

	General coefficient CECS		Anger suppression		Depression suppression		Anxiety suppression	
	Mean value ±SD	*p* value	Mean value ±SD	*p* value	Mean value ±SD	*p* value	Mean value ±SD	*p* value
Vaccination against COVID-19								
Yes (*n* = 146)	53.07 ± 11.94	0.2	16.68 ± 5.05	0.4	18.17 ± 4.11	**0.04**	18.22 ± 4.90	0.4
No (*n* = 448)	51.43 ± 12.33		16.26 ± 5.18		17.32 ± 4.57		17.85 ± 5.01	
Willingness to vaccinate at the earliest convenience								
Yes (*n* = 269)	52.37 ± 12.44	0.2	16.62 ± 5.19	0.2	17.59 ± 4.71	0.1	18.16 ± 5.02	0.2
No (*n* = 88)	50.41 ± 13.81		16.50 ± 5.33		16.59 ± 5.10		17.32 ± 5.49	
I do not know (*n* = 91)	50.68 ± 10.36		15.62 ± 4.94		17.33 ± 4.80		17.73 ± 4.39	
Wearing a mask in indoor public places								
Always or almost always (*n* = 556)	51.83 ± 12.16	0.9	16.37 ± 5.20	0.9	17.55 ± 4.69	0.6	17.92 ± 4.95	0.7
Sometimes or occasionally (*n* = 38)	51.63 ± 14.01		16.16 ± 4.41		17.05 ± 5.49		18.42 ± 5.59	
Wearing a mask in outdoor public places (streets, parks etc.)								
Always or almost always (402)	52.06 ± 11.87	0.3	16.47 ± 5.11	0.2	17.49 ± 4.64	0.8	18.10 ± 4.71	0.3
Sometimes or occasionally (140)	51.93 ± 12.83		16.60 ± 5.24		17.87 ± 4.72		17.46 ± 5.39	
Never or almost never (52)	50.40 ± 13.65		15.12 ± 5.02		16.76 ± 5.67		18.52 ± 5.66	
Keeping distance in outdoor public places								
Always or almost always (314)	51.72 ± 12.78	0.2	16.39 ± 5.15	**0.03**	17.38 ± 4.81	0.7	17.94 ± 5.05	0.4
Sometimes or occasionally (196)	53.02 ± 10.88		17.03 ± 5.16		17.93 ± 4.44		18.06 ± 4.74	
Never or almost never (84)	49.85 ± 13.02		14.80 ± 4.78		17.07 ± 5.19		17.98 ± 5.23	
Keeping distance in indoor public places								
Always or almost always (376)	52.06 ± 12.72	0.9	16.54 ± 5.06	0.7	17.52 ± 4.73	0.7	18.00 ± 5.14	0.7
Sometimes or occasionally (184)	52.03 ± 13.09		16.65 ± 5.09		17.34 ± 4.69		18.04 ± 4.48	
Never or almost never (34)	51.27 ± 11.23		15.92 ± 5.38		18.47 ± 5.21		16.88 ± 6.01	
Hand disinfection before entering public facilities								
Always or almost always (350)	53.97 ± 14.37	0.4	17.23 ± 6.35	0.2	18.67 ± 5.24	0.3	18.08 ± 6.02	0.8
Sometimes or occasionally (166)	51.95 ± 11.99		16.54 ± 5.04		17.33 ± 4.67		18.09 ± 4.59	
Never or almost never (78)	50.53 ± 11.75		15.54 ± 4.69		17.38 ± 4.61		17.60 ± 5.31	
Disinfection or leaving purchased goods in “quarantine”								
Always or almost always (108)	54.02 ± 12.69	0.2	17.07 ± 5.10	0.4	18.04 ± 4.86	0.2	18.91 ± 4.67	0.2
Sometimes or occasionally (162)	50.00 ± 12.33		15.90 ± 5.03		16.80 ± 5.04		17.30 ± 4.67	
Never or almost never (324)	52.00 ± 12.02		16.34 ± 5.23		17.70 ± 4.52		17.96 ± 5.22	

SD – standard deviation; Statistically significant differences have been marked in bold.

The average perceived stress level in the studied group was 20.5 ± 5.3. The level of perceived stress did not differentiate the general coefficient of emotional control (*R* = −0.022912; *p* = 0.6), anger suppression (*R* = −0.078530; *p* = 0.05), depression suppression (*R* = 0.055603; *p* = 0.2) and anxiety suppression (*R* = −0.033698; *p* = 0.4).

In the test of knowledge about COVID-19 and measures of its prevention, the average number of points scored was 7.29 ± 1.59. More than half of participants (56.9%) scored 8 and 9 points in the knowledge test. The average level of knowledge on these aspects characterized 36.4% of participants of the study, and four or less points were obtained by 6.7% of the participants. Statistical analysis revealed that the higher level of knowledge, the higher value of the general coefficient of emotional control and the anger and anxiety suppression indexes. *Post hoc* tests indicated the existence of statistically significant differences in the general coefficient of emotional control between people with low level of knowledge and respondents with high level of knowledge about COVID-19 (45.67 ± 11.76 vs. 52.95 ± 11.63). People with low level of knowledge were characterized by low suppression of anger and anxiety (14.43 ± 5.67 and 15.09 ± 3.60, respectively) compared to people with average and high knowledge. The Spearmann rho correlation coefficient between the general coefficient CECS and knowledge about COVID-19 was 0.2318 at *p* = 0.001, for the subscale of anger suppression: *R* = 0.2336 at *p* < 0.0001, and for the subscale of anxiety suppression: *R* = 0.2169 at *p* = 0.003. The analysis showed no correlation between the level of knowledge and the depression suppression subscale. Detailed data are presented in Table 5.

**Table 5:** Level of emotional control in the studied group and knowledge on COVID-19 pandemic.

Level of COVID-19 knowledge	General coefficient CECS		Anger suppression		Depression suppression		Anxiety suppression	
	Mean value ±SD	*p* value	Mean value ±SD	*p* value	Mean value ±SD	*p* value	Mean value ±SD	*p* value
Low (≤ 4 points) (*n* = 40)	45.67 ± 11.76	**0.02**	14.43 ± 5.67	**0.03**	16.14 ± 5.39	0.09	15.09 ± 3.60	**0.02**
Average (5–7 points) (*n* = 216)	51.45 ± 12.87		15.97 ± 5.09		17.22 ± 4.85		18.26 ± 5.36	
High (8–9 points) (*n* = 338)	52.95 ± 11.63		16.92 ± 5.07		17.94 ± 4.52		18.09 ± 4.74	

Correlation

COVID-19 knowledge – general coefficient CECS: *R* = 0.232; ***p* = 0.001**.

COVID-19 knowledge - anger suppression: *R* = 0.234; ***p* = 0.0009**.

COVD-19 knowledge – depression suppression: *R* = 0.167; *p* = 0.054.

COVID-19 knowledge – anxiety suppression: *R* = 0.217; ***p* = 0.004**.

SD – standard deviation; Statistically significant differences have been marked in bold.

## Discussion

The aim of this study was to examine the level of emotional control in a group of adult Poles during the third wave of the COVID pandemic and the restoration of many anti-epidemic restrictions. Previous studies on emotional control during the pandemic were conducted in a group of healthcare professionals and nursing students. However, these surveys did not include the general population. Therefore, similar analysis conducted in the group of general population may shed a new light upon the current state of knowledge in this area of research.

In a study by Bidzan et al. (2020) which was conducted in the first days after the declaration of a pandemic in Poland among hospital employees, the average level of emotional control was 49.74. In turn, in both studies by Malinowska-Lipień et al. (2021a,b), conducted among nurses during the second wave of the pandemic in Poland, the average level of emotional control was much higher and oscillated around 54. On the other hand, the average level of emotional control in a group of nursing students, in a study conducted at a similar time as research described in the present study, was about 51points in Poland, 51.40 in Spain and 52.69 in Slovakia (Kupcewicz et al., 2022a,b). The result obtained in this study (51.82) indicated an average level of emotional control and was comparable to the above-mentioned studies. However, the obtained result was slightly higher than the values obtained during the standardization tests of the CECS questionnaire, which were carried out in 1998 in the general population (Juczyński, 2012). In those studies, the average general coefficient of emotional control for women was 49.97, and for men 51.42 (in this study: 51.34 and 53.6, respectively). The higher value of the general coefficient of emotion control in this study, compared to normalization studies, resulted primarily from a slightly greater suppression of depression in both genders (women: 17.41 vs. 16.88, men: 17.92 vs. 16.85) and anger by men (17.19 vs. 16.19). The level of anxiety suppression during both studies was at a similar level. Existing differences in emotion control between genders, similarly to normalization studies but opposite to study by Malinowska-Lipień et al. (2021b), were not statistically significant.

The results of normalization studies clearly indicated the intensification of subjective control of all three emotions with age (Juczyński, 2012). This conclusion is only partially consistent with the results obtained in this study which showed that the lowest suppression of anger and depression was found in people aged 30–39 years old. It is probably related to having small children, and the need to combine remote work with looking after them when nurseries and kindergartens were closed (which took place during the study). Respondents reporting difficulties in combining these two duties were significantly less likely to inhibit the feeling of anger. Remote work itself did not translate into differences in emotional control. Low suppression of anger in this particular group of people is not surprising. Anger is a feeling that can provide a sense of control. The individual can at least blame others (e.g., government), which is a state of mind that may be preferred to uncertainty: not knowing what will happen next (Abadi et al., 2021). The studies conducted so far clearly indicated that restrictions in the form of a deep lockdown accompanied by the transfer of parents to remote work from home and children to home (remote) learning have a deep and complex impact on families. Parenting challenges are compounded by the demands of working from home, economic hardships and layoffs, and social restrictions imposed on parents. It is believed that the profound changes in daily family life caused by the pandemic may fuel parental stress and family tensions (Clemens et al., 2020; Cluver et al., 2020; Calvano et al., 2022). In order to prevent this, it is necessary to develop effective and tailored family support programs, so that stress and emerging negative emotions do not find an outlet in the form of aggressive, violent behavior (Prokupek et al., 2023).

Similarly to results obtained by Malinowska-Lipień et al. (2021a) it was not found that the COVID-19 infection modified the level of emotional control. Out of the experiences related to the pandemic, only the death of a loved one due to COVID-19 significantly modified emotional control. The death of a loved one is one of the strongest life stressors. People who experienced the death of loved ones are characterized by a significant deterioration in physical and mental well-being and social functioning, which may persist up to 4 years after the loss of a loved one (Liu et al., 2019).

The level of emotional control was positively correlated with the level of knowledge about COVID-19 and its preventive measures. Knowledge and awareness of the threats caused by COVID-19 (e.g., by experiencing the death of a loved one due to COVID-19) enhances greater suppression of emotions. Lack of knowledge translates into a lack of understanding of the introduced restrictions and their long maintenance (Miller et al., 2021; Rahman et al., 2022). This results in a lower level of emotional control which may be a reason to undermine the introduced restrictions and not comply with them. In the conducted research, it was noted that there was a tendency to lower suppression of emotions by people who never or almost never complied with the mandatory restrictions, especially in terms of keeping distance and hand disinfection, with statistically significant differences found only in the case of anger suppression depending on keeping distance in outdoor public places.

Controlling emotions also means being emotionally correct and it is a trait of highly socialized and highly educated individuals who rigidly adhere to social norms. Greater emotional control among people with higher knowledge may not mean suppressing them, but rather making an effort to reduce negative emotions appearing in a stressful situation, i.e., it is a manner of coping with stress. In other words, emotional arousal is slightly suppressed in order to take more constructive actions to change the stressful situation (Averill, 2004; Kappas, 2013; Janowski et al., 2014; Liu et al., 2023). In the case of pandemic, it may mean complying with the imposed restrictions in order to deal with the threat faster and return to normal life. It may also explain the lack of correlation in the studied group between the level of experienced stress and the level of emotional control. For some of the respondents, the reaction to high levels of stress will be expressing emotions (low suppression), for others inhibiting them. This suggestion, of course, needs to be verified in subsequent studies. In this study, strategies for coping with stress were not tested, and only such a study could clearly explain the observed relationships. Nevertheless, it seems that educational campaigns should be organized to explain in a simple, straightforward and calm way the threats related to the spread of the SARS-CoV-2 virus and the reasons for introducing certain restrictions. Increasing knowledge on this aspect may improve emotional control, not in the context of suppressing emotions, but rather as a way of coping with a difficult situation.

However, it is worth to acknowledge some potential limitations of the study. First, the research relies on self-report measures, which may introduce response biases and potential inaccuracies. Additionally, the study included only the adult population in Poland. It may affect the overall representativeness of the results. Limitations of the study include also the overrepresentation of people with high educational level and women among respondents. It should be noted that the study was conducted in the form of an online survey. This way of conducting research has some disadvantages that should be taken into account and which may affect the representativeness of the results too. These include, among others, exclusion from the study of people without access to the Internet, therefore the study includes people who are relatively wealthy, with higher than average knowledge on technology, and younger. Older people have less access to the Internet or are less familiar with it than younger people. Similarly, people with lower levels of education also have lower Internet access and computer skills. For this reason, the results and conclusions drawn from this study relate only to the studied group of respondents. There is no possibility of generalizing the obtained results onto the entire population (country or region), other contexts or age groups. However, both the results and the conclusions of the study can be treated as signal information, providing a basis for conducting similar studies in larger groups.

Moreover, many other factors may influence the emotional control. These factors include: type of personality, level of sociability, existence of mental disorders, strategy to cope with stress and physical activity (Janowski et al., 2014; Gogola et al., 2021; Markofski et al., 2022). These factors were not analyzed in this study. However, their potential impact on the observed results should be taken into account.

## Conclusion

Proper education aimed at improving knowledge about COVID-19 and methods of prevention may enhance the control of emotions in the population. Possible future preventive measures aimed at limiting the spread of SARS-CoV-2 infections or other infectious diseases should also take into account possible excessive mental burden caused by private and professional duties.

## Data availability statement

The raw data supporting the conclusions of this article will be made available by the authors, without undue reservation.

## Ethics statement

Ethical review and approval was not required for the study on human participants in accordance with the local legislation and institutional requirements. Written informed consent for participation was not required for this study in accordance with the national legislation and the institutional requirements.

## Author contributions

All authors listed have made a substantial, direct, and intellectual contribution to the work and approved it for publication.

## Conflict of interest

The authors declare that the research was conducted in the absence of any commercial or financial relationships that could be construed as a potential conflict of interest.

## Publisher’s note

All claims expressed in this article are solely those of the authors and do not necessarily represent those of their affiliated organizations, or those of the publisher, the editors and the reviewers. Any product that may be evaluated in this article, or claim that may be made by its manufacturer, is not guaranteed or endorsed by the publisher.
